# The role of the cultural environment in the development of physical literacy and physical activity of Iranian children

**DOI:** 10.1186/s12887-023-04297-3

**Published:** 2023-09-20

**Authors:** Majid Mohammadi, Farzaneh Elahipanah, Sadegh Amani-shalamzari

**Affiliations:** 1 Department of Motor Behavior and Sports Psychology, Faculty of Physical Education and Sports Sciences, University of Tehran, Tehran, Iran; 2 Department of Sport Management and Motor Behaviour, Faculty of Physical Education and Sports Science, Kharazmi University, Tehran, Iran; 3 Department of Exercise Physiology, Faculty of Physical Education and Sports Science, Kharazmi University, Tehran, Iran

**Keywords:** Canadian Assessment of Physical Literacy Test-2, The International Physical Activity Questionnaire, Geographic location, Place of residence

## Abstract

**Background:**

The investigation of physical literacy (PL) and physical activity (PA) should be done in an ecological context because the socio-cultural situation can encourage or inhibit children’s activity. The present study aimed to study the role of the cultural environment in the development of PL and PA in Iranian children.

**Methods:**

The statistical population consisted of Iranian children aged 8 to 12, of whom 270 students were recruited by cluster sampling from six provinces. They complete the Canadian Assessment of Physical Literacy 2 (CAPL-2) and the International Physical Activity Survey. To examine the cultural environment, the components of geographic location (west, south, central, east, and northwest), place of residence (downtown, suburban, and village), and gender (boys and girls) were used. A multivariate ANOVA analysis was used to analyze the data.

**Results:**

From the geographic location, the findings showed that students inhabit in the west and east of Iran have significantly higher scores than their counterparts living in the central, south, and northwest at PA and PL (P < 0.001). From the place of residence, we observed a higher level of PA and PL in children living in the village than in those living in the suburbs and downtown (P < 0.001). In terms of gender, boys have higher PL and PA scores (P < 0.001).

**Conclusion:**

The findings demonstrate that socio-cultural factors, geographic location, place of residence, and gender have different impacts on children’s PL and physical activity. Therefore, we suggest using ecologically appropriate decentralized planning in a physical education curriculum.

## Introduction

Physical activity (PA) has lifelong health benefits, and being active in childhood is particularly important because children’s active lifestyles are shifted into adulthood. Sedentary behaviors such as watching screens are associated with an increased risk of childhood obesity, hypertension, and mortality [1]. In many countries, measures have been taken to promote PA and understand potential solutions for health and wellness in childhood and adolescence [2]. To achieve physical and mental health and well-being, the World Health Organization (WHO) recommends that persons aged 5 to 18 years have 60 min of moderate/intense PA per day [3]. Through common measures, European countries have declared regular PA as an effective preventive action to reduce risk factors for health in all ages, ethnicities, and socio-economic groups [4]. However, more than half of children aged 6 to 11 years do not follow the recommended amount of PA [4], and a sedentary lifestyle during childhood can affect the quality of life of adolescents and adults [5]. In this regard, the multidimensional construction of physical literacy (PL) has been recognized as an important prerequisite for PA and exercise [6, 7].

According to Whitehead’s opinion [6], PL is related to the ultimate goal of a qualified physical education program, including motivation, self-confidence, physical competence, knowledge, and perception of maintaining PA throughout life. PL has many benefits, including improved health care and physical and mental health, increased efficiency in day-to-day activities, skills development, and increased participation in sports [8]. Cultivating these aspects enhances the experience and contributes to the realization of all human potential [6]. In addition, the person acts with high intelligence and focuses on the aspects of perception of the physical environment, anticipating the need for movement and meeting it appropriately [9]. PL is becoming the guiding ideology for the promotion of PA in educational institutions to maximize health benefits [10]. PL is affected by the cultural context, and the social and physical circumstances in which children grow up, therefore the children’s PL level is different in countries [11–13]. As a result, understanding the causes of these differences leads to the development of effective interventions to improve the health and well-being of children [14] because, with the change of residence, the social-cultural-economic status affects the child’s motor development changes.

Socio-cultural facilities and attitudes can prevent and encourage PA and exercise [15]. The richer the sports facilities of the region and the general culture of the residents, the higher PL of the children will be due to doing more physical activities. Based on developmental context theory (environmental context theory), which refers to the mutual dynamic relationship between a growing person and the changing environment in which she/he lives, residency and culture can influence children’s PA and PL, because there are multi-dimensional correlations between child characteristics, sociocultural factors, and geographic location with motor behaviors [16]. In this regard, Super [17] stated that cultural practices lead to the development of gross movements in Kipsigis babies in western Kenya (Africa). Additionally, Bril et al. [18], reported also slow growth of gross motor skills in children of Western cultures. Furthermore, cultural factors significantly influence FMS scores among Asian and European children between the ages of 9 and 11 [19]. The cultural context has also been shown to influence the relationship between physical fitness levels between two Asian countries [20] and motor competence between two neighboring nations on the same continent [21]. Therefore, the PL and PA study of children should be conducted based on the ecological environment, because the possible differences between children’s PA and PL can provide a better perception of different cultural settings for the development of motor skills.

Different ethnic groups may have different cultural criteria for developing movement skills depending on the different regions they reside in. Considering that there are various ethnic groups with different cultures in Iran, to achieve the desired PA levels in the country, the level of PL and PA in cities needs to be determined for effective strategies and programs to be developed. Also, every region in Iran is pre-eminent in a special sport and therefore it seems that the geographical region and native culture of that region play a role in this situation. Therefore, this study was intended to study the role of the cultural environment on the development of PL and PA among Iranian children, hopefully, the findings will help develop effective physical training programs and remove barriers to encourage lifelong PA. Some of the constraints are religious problems in exercising girls, problems in schools in physical education lessons such as the place of exercise, having a sports teacher, and time devoted to physical education.

## Materials

### Participants

**Fig. 1:**
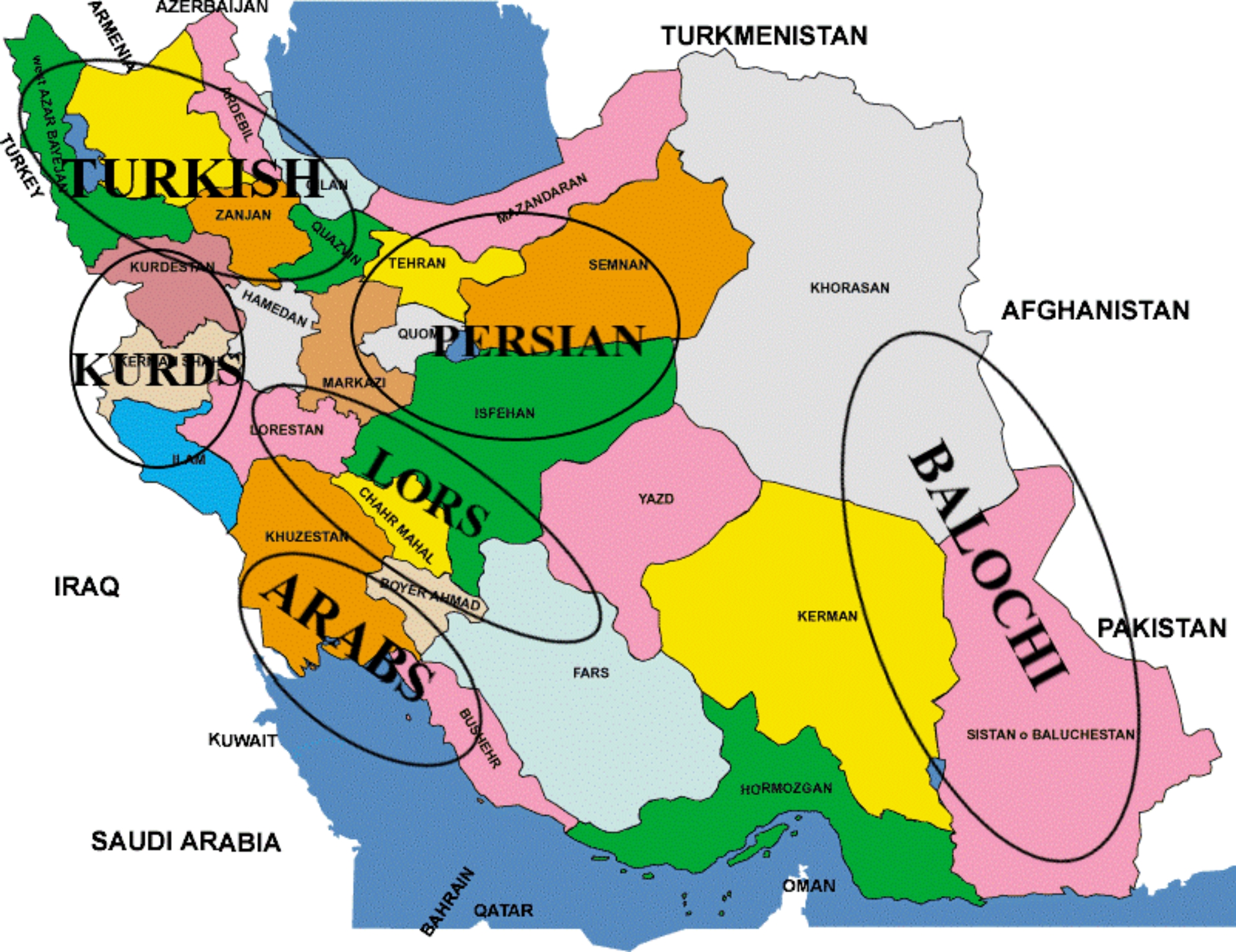
The location of major ethnicities of Iran

Field-based causal-comparative research was conducted. A convenient multi-stage cluster sampling method was implemented to select the statistical sample. Participants were 270 students aged 8–12 years from six from six main Iranian ethnicities. Initially, six provincial centers with different ethnic groups, including Khorramabad (Middle West, Lors), Ahvaz (South, Arabs), Tehran (Central, Persian), Sanandaj (West, Kurds), Zahedan (East, Balochi), and Tabriz (Northwest, Turkish) were selected based on ethnic and geographical location. Figure 1 shows the distribution of ethnic groups in different regions of the map of Iran. Then within each center, three blocks representing the residence (city, suburb, and village) were chosen. In the following step, 15 students were randomly selected for each block, so that there are 45 subjects in each province, (city center: 15 n, suburbs: 15 n, village: 15 n). It should be noted that the blocks were chosen through unequal probabilistic sampling or disproportionate sampling. Additionally, data collection occurred during the corona outbreak, schools were closed and it was difficult to select more subjects.

### Data collection

The survey provided the individual characteristics of the participants. PL was measured with the substructures of the CAPL-2 model which include daily physical activity, physical competence, motivation, self-confidence, knowledge, and perception. The four CAPL-2 domains were assessed according to the CAPL-2 manual for measuring PL levels. Daily PA was determined through two tests: (a) direct measurement of physical activity using a pedometer for one day (waking to sleep at night) and then in one week; (b) Indirect measurement of physical activity using a PL questionnaire; The purpose of this test is to assess children’s perception of their participation in daily activities at a moderate to high intensity and a minimum volume of 60 min. The total score for this domain is 30 points, 25 points for the pedometer, and 5 points for self-reporting of physical activity by the child [22]. Physical competence included three criteria: (a) the 15-20 m Progressive Aerobic Fitness Test (PACER) to assess aerobic fitness [23]; (b) the Plank trunk test to evaluate muscle endurance [24]; And (c) the Canadian Agility and Movement Skill Assessment (CAMSA) [25] that is a realistic measurement of selected complex and combined fundamental movement skills, such as catching, throwing and jumping. The total score for the physical competence domain was 30 points, 10 points for PACER, 10 points for Plank, and 10 points for CAMSA. The area of motivation and confidence assesses children’s confidence in their ability to be physically active and their willingness to participate in PA. This domain is conceived in four components: desire, suitability, competence, and internal motivation, the maximum score for each of these domains was 7.5 and the total score was 30. The knowledge and perception domain assessed the child’s knowledge of PA through five questions [26]. The total score in this area was 10 points. The point range for questions 1 to 4 was zero to one and for question 5 it was zero to six. The total PL score is 100 points. CAPL-2 has quantitative and qualitative scores [27]. The total PL score is calculated based on the quantitative scores obtained for each subscale, item, and their sum and the classification of scores in four qualitative levels, from one to four, including basic level (< 43), progression (43.8 to 63.8), achievement (63.8 to 74) and excellence (> 74) [22]. The validity of the CAPL-2 test to measure the PL level of Iranian children has been culturally confirmed [28].

In addition, the International Physical Activity Questionnaire (IPAQ) for Children was used to assess general levels of PA throughout the primary school year for 8-14-year-olds. The questionnaire contains 10 questions that measure the overall level of moderate to high PA from the previous week [29]. Nine questions are recorded on a Likert scale from 1 to 5. The response that indicates the least amount of PA is assigned to one score and the response that indicates the most amount of PA is assigned to five scores. Question 10 is only intended to identify students who have an unusual activity during the previous week and therefore does not count toward the individual score. In this questionnaire, total scores between 1 and 2.33 are ranked as low, scores between 2.34 and 3.66 as moderate, and scores above 3.67 to 5 as high PA level [29]. According to a study of Iranian children, the reliability of this questionnaire was found to be 0.89 using the Cronbach alpha method [30].

### Statistical analysis

The Statistical Package of Social Sciences (SPSS, IBM, v23) was used to analyze data. Data presented in mean ± standard deviation (SD). At first, assumptions of normality of data (Kolmogorov-Smirnov test), homogeneity of variance (M box), and homogeneity of regression slopes were confirmed. Multivariate analysis of variance and one-way analysis of variance was performed to analyze the data. If required, the Bonferroni post hoc test was used. The minimum and maximum age of participants was between eight and 12. The Kolmogorov-Smirnov test at a confidence level of 95% confirmed the normal distribution of the data. In addition, the Mbox test results were equal to (P = 0.235), so Wilks’ Lambda values are reported. The significance level was considered at p ≤ 0.05 for all statistical analyses.

## Results

The results showed that the amount of PA and PL in Iranian children is medium and increasing (Table 1). In addition, the PA and PL levels of children living in the village were higher than their suburban and downtown counterparts. Furthermore, the results showed that boys scored higher than girls on both of these indicators.

**Table 1:** Quantitative and qualitative outcomes of physical activity and physical literacy of participants (mean ± SD)

Variable	Indicator	Physical activity		Physical literacy	
		Mean ± SD	Qualitative level	Mean ± SD	Qualitative level
Geographic regions	Ahvaz (South)	2.74 ± 0.74	Medium to low	55.41 ± 1.19	Progressing
	Khorramabad (West)	3.04 ± 0.68	Medium	59.60 ± 1.32	Progressing
	Sanandaj (West)	3.10 ± 0.67	Higher than average	60.12 ± 1.41	Progressing
	Tehran (center)	2.58 ± 0.66	Medium to low	52.17 ± 2.20	Progressing
	Tabriz (North-west)	2.81 ± 0.63	Medium to low	56.77 ± 1.36	Progressing
	Zahedan (East)	3.16 ± 0.72	Higher than average	60.19 ± 1.80	Progressing
Place of residence	City center	2.62 ± 0.49	Medium to low	52.23 ± 0.97	Progressing
	Suburb	2.81 ± 0.52	Medium	54.33 ± 1.09	Progressing
	Village	3.13 ± 0.66	Higher than average	61.95 ± 0.91	Progress towards achieving
Gender	Boy	3.05 ± 0.47	Medium	59.07 ± 0.85	Progressing
	Girl	2.68 ± 0.49	Medium to low	53.24 ± 0.48	Progressing

In daily physical activity, physical competence, motivation, and self-confidence of children living in the west and east of the country have better outcomes. However, in knowledge and perception, children living in central and northwest areas scored higher (Table 2).

**Table 2:** Physical literacy components of participants (mean ± SD)

Variable	Indicator	Daily physical activity (30 points)	Physical competence (30 points)	Motivation and self-confidence (30 points)	Knowledge and perception (10 points)
Geographic regions	Ahvaz (South)	17.30 ± 0.67	12.23 ± 0.42	19.32 ± 0.74	5.36 ± 0.74
	Khorramabad (West )	18.90 ± 0.54	14.26 ± 0.44	21.78 ± 0.32	5.25 ± 0.45
	Sanandaj (West)	19.10 ± 0.74	13.33 ± 0.43	19.46 ± 0.54	4.97 ± 0.21
	Tehran (center)	14.77 ± 0.54	9.84 ± 0.23	17.97 ± 0.33	6.01 ± 0.31
	Tabriz (Northwest)	17.87 ± 0.42	13.30 ± 0.54	19.78 ± 0.35	5.71 ± 0.45
	Zahedan (East)	20.15 ± 0.46	13.74 ± 0.72	21.62 ± 0.62	4.60 ± 0.32
Place of residence	City center	16.06 ± 0.47	11.44 ± 0.34	18.80 ± 0.38	5.93 ± 0.25
	Suburb	17.70 ± 0.48	12.09 ± 0.44	19.96 ± 0.25	4.78 ± 0.24
	Village	20.89 ± 0.46	14.84 ± 0.37	22.23 ± 0.19	4.12 ± 0.22
Gender	Boy	19.37 ± 0.42	13.82 ± 0.33	20.75 ± 0.62	4.90 ± 0.18
	Girl	17.05 ± 0.40	11.75 ± 0.33	19.90 ± 0.56	4.71 ± 0.12

Table 3 demonstrates the significant effect of geographic location, place of residence, and gender, as well as the interaction of geographic location-gender and place of life-gender. It shows that there is a significant difference between the amount of PA and the level of PL of the participants.

**Table 3:** The results of the multivariate analysis of variance test to examine physical activity and physical literacy of Iranian children according to geographic regions, place of living, and gender

Variable	Effect	Landai Wilk	Def	F	Sigh	ETA squared
Geographic regions	Geographic regions	0.315	5	6.395	0.001**	0.439
	Gender	0.648	1	3.734	0.004**	0.314
	Geographic regions × Gender	0.644	5	2.012	0.021**	0.194
Place of residence	Geographic regions	0.364	2	6.851	0.001**	0.206
	Gender	0.194	1	5.839	0.001**	0.102
	Geographic regions × Gender	4.203	2	2.775	0.001**	0.068

Table 4 shows that there is a significant difference between PL and its components and the amount of PA in Iranian children in terms of geographic location. To accurately determine the difference, Bonferroni’s post hoc test was performed; the results showed that children living in the west and east of Iran, have better scores in daily physical activity, physical competence, motivation, and self-confidence, and total PL and PA (P < 0.001). With the knowledge and perception components, participants living in the central and northwest had higher scores.

Moreover, a significant difference was observed between PL and its components as well as the amount of PA in Iranian children in terms of place of residence. From the amount of PA and PL, children living in the village have higher scores than their counterparts living in the central and suburbs (P = 0.001), but there was no significant difference between children living in the center and the suburbs (P = 0.145).

**Table 4:** The results of the one-way analysis of variance test to compare physical literacy and physical activity of Iranian children

Variable	Indicator	Sum of squares	Def	Mean square	F	Sigh	ETA squared
Geographic Regions	Daily physical activity	794.651	5	158.930	19.929	0.001**	0.241
	Physical competence	528.677	5	105.735	21.068	0.001**	0.253
	Motivation and self-confidence	479.930	5	95.986	19.424	0.001**	0.238
	Knowledge and perception	115.121	5	23.024	10.303	0.001**	0.142
	Physical literacy	367.179	5	73.465	22.41	0.001**	0.261
	Physical activity	10.633	5	2.321	10.717	0.001**	0.147
Gender	Daily physical activity	232.067	1	232.067	17.831	0.001**	0.248
	Physical competence	52.267	1	52.267	8.943	0.004**	0.142
	Motivation and self-confidence	3.033	1	3.033	0.502	0.482	0.009
	Knowledge and perception	1.067	1	1.067	0.366	0.548	0.007
	Physical literacy	649.380	1	649.380	11.704	0.001**	0.178
	Physical activity	2.356	1	2.356	13.150	0.001**	0.196
Place of residence	Daily physical activity	773.023	2	386.511	28.447	0.001**	0.231
	Physical competence	418.897	2	209.441	23.425	0.001**	0.199
	Motivation and self-confidence	395.766	2	197.883	23.07	0.001**	0.196
	Knowledge and perception	108.740	2	54.370	14.63	0.001**	0.134
	Physical literacy	3364.207	2	1682.103	29.59	0.001**	0.238
	Physical activity	7.194	2	3.597	18.49	0.001**	0.213
Gender	Daily physical activity	253.851	1	253.851	18.68	0.001**	0.169
	Physical competence	204.16	1	204.16	22.83	0.001**	0.181
	Motivation and self-confidence	32.901	1	32.901	2.21	0.086	0.090
	Knowledge and perception	10.821	1	10.821	2.19	0.089	0.080
	Physical literacy	1610.490	1	1610.490	28.33	0.001**	0.213
	Physical activity	6.54	1	6.54	33.60	0.001**	0.242

Regarding the knowledge and perception components of PL, children living in central and suburban areas of the city scored higher than children in the village, but in other parts, children living in the village had superior points. Also, in terms of gender, there was a significant difference between boys and girls in general PL (P = 0.001), PA (P = 0.001), physical competence (P = 0.004), daily PA (P = 0.001) boys had higher scores in these components. However, in the motivation and self-confidence component (P = 0.481) and knowledge and perception (P = 0.548), no gender difference was found between the participants.

## Discussion

This research was done to study the role of the cultural environment in the development of PL and PA in Iranian children. The findings have shown that Iranian children generally lack optimal levels of PA and PL. In other words, the level of PL of Iranian children aged 8 to 12 was progressing qualitatively towards the beginner level, and in terms of PA, it was of medium to low quality at best. Fundamental steps must therefore be taken in this field.

The lack of an acceptable quality level of PL among Iranian children was not an unexpected finding. This finding was already supported by the research of Valadi et al. Who reported that students in the second to sixth grades in Tehran (8–12 years) do not have an acceptable level of PL [28]. In fact, according to the concept of PL, this study shows that elementary school students in Iran are far from having an active lifestyle and a healthy lifestyle. This could be related to the short hours of physical education classes at Iranian schools, which are less than 120 min per week. Unfortunately, in recent years, physical education classes for grades 1 to 3 have been eliminated. Also, in the metropolis, there are few sports spaces available for children and they have to play virtual games in small apartments. This problem may expose the health of this segment of society to various physical, mental, and mobility risks. Lifestyle changes and inactivity have resulted in children today failing to meet recommended physical activity guidelines. Consequently, children are in poor physical condition. Since the foundations of an active lifestyle are formed during childhood. It is suggested that the education system increase the number of hours of physical education to promote PA and PL.

In the geographic location, a significant difference was observed in Iranian children with PA and PL. Regarding the components of daily PA, physical fitness, motivation, and self-confidence, participants living in the west (Khorramabad, Sanandaj) and east (Zahedan) have significantly higher scores than their counterparts living in the central (Tehran), southern (Ahvaz) and northwestern (Tabriz) regions. In the substructure of knowledge and perception, participants living in the central and northwest had higher scores than other regions. Tahmasabi et al. Also pointed out in movement skills, seven to nine-year-old children living in western Iran had a higher score than children living in the center and south [15]. Among the possible reasons, it may be mentioned that children have more activity and space in the western and eastern areas of Iran than in the central and southern areas. Today, environmental conditions and technological advances in metropolises and large cities have left children inactive. Children in large cities face restrictions in their houses and neighborhoods, furthermore, the increase in the number of students in schools and the lack of sufficient space has reduced practical opportunities for children. Children in metropolises of Ahvaz, Teheran, and Tabriz, because of their smaller living space, are less able to engage in activities that involve their large muscles. They are most active in the virtual space and perform activities that involve fine muscles. On the other hand, children in the cities of Sanandaj, Khorramabad, and Zahedan are more likely to participate in activities that involve their big muscles as a result of their larger living environment. In these activities, children’s fundamental skills are reinforced, resulting in improved levels of PL and physical activity. Another possible explanation of the difference in PL and PA scores among students from different regions is consistent with development context theory. From this perspective, the dynamic relationship between growing people and the changing environment in which they live is considered to be the basis of developmental and behavioral changes [31]. The environment and environmental conditions affect the behavior of the child, the cultural environment in which we live and issues such as beliefs, customs, and attitudes affect the motor development of the child [31]. It appears that geographic areas with different features create different opportunities for children to engage in sports and recreation, and these factors lead to differences in children’s movement levels. According to the results of this study and the previous one [15], based on the different effects of geographical location on the movement pattern of children, it is appropriate that the planning of the Iranian Ministry of Education be developed at the regional level and taking into account the particular cultural factors of each region. In the centralized education system in Iran, curricula, objectives, allocation of teaching time, and student evaluation methods and policies related to credit allocation and staffing are developed at the national level and the teacher is the executor and facilitator of the developmental programs. This is even though the educational system is decentralized in most of the leading countries.

Our findings showed the effect of the place of life (city, suburb, and village) on the level of PA and PL of Iranian children were different. In this regard, the results showed that the level of PL of children living in the village was higher than that of children in the center and the suburbs. Regarding the components of daily PA and physical competence, which are more related to the amount of PA and PL, the results showed that the children living in the village are in a better condition. More activity and space for children in the village than in the city is a potential reason for this observation from physical space. Private schools in the cities mostly use buildings whose spaces are not reserved for schools and which have been converted to schools with little modification. There is little available space in public schools, or crowding in schools prevents the proper use of space. Meanwhile, in the village, the school space is suitable for play and physical activity. Furthermore, urban students are transported to schools by taxi and car, but rural students walk to school; as well, in nearby villages, there is generally one school where students have to walk a long distance. One study found that children in rural areas had better movement test scores than their counterparts in urban areas [32]. In this regard, Tsapakidou et al. [33] compared the motor skills of children aged 8 and 9 according to their socio-economic status and place of residence. Consistent with the results of our research, they also indicated that the location of residence affected the quality of fundamental motor skills. In terms of knowledge and perception, the results demonstrated better performance of children in the center and the suburbs than children in the village. One of the reasons for the low level of knowledge and perception is that, in Iran’s education system, less attention is paid to the exercise training needed for this group. As a result, students did not have regular, organized physical activities that would benefit their present and future, or they had fewer such programs. Therefore, in addition to further supervision of intra-school physical activities that are related to physical competence, the teaching of cognitive concepts should be done in physical education lessons. Practical definitions of physical education, parameters of physical fitness, hygiene, and health, and the side effects of over-use of screen-compatible programs are important concepts, which should be taught in primary schools, especially in rural schools. We recommendthat schools in the metropolises, plan sports programs for students outside of school time to promote the level of PA.

Determining the relationship between the level of PL with age and gender can lead to the initial recognition of patterns among this age group of society [22]. On this basis, it was found that there is a significant difference between the two genders in the PA and PL levels, so the boys were better than the girls. Its cause may be partly related to religious and cultural restrictions in Iran, which makes girls unable to enjoy physical activity as much as boys. The lack of suitable space and facilities, the attitude of families as well as the low participation of girls in physical education in schools, lead to their low level of movement compared to boys. Moreover, the Victorian effect on the negative effect of exercise on women’s fertility still exists in some cities and in particular in the villages of Iran, and in many cases, it denies girls the opportunity to participate in many dynamic sports. It seems that in western societies, gender is not the cause of the difference in PL of boys and girls. VedulKjelsas et al. reported an insignificant difference between the motor skills of 11-year-old Norwegian boys and girls, which was associated with slight male superiority [34]. Also, Goodway et al. reported that there is no significant difference in boys’ and girls’ motor skills in the Midwest and Southwestern America [35]. American girls have more freedom to act and more athletic opportunities. Ball games, track and field, dance, and gymnastics are the core content of the physical education program in the United States. However, in Iran, the physical education program consists of two hours per week, with no specific content. Add to this deplorable situation, fewer sports facilities for girls in schools. Our recommendation is that in addition to increasing physical education hours, there should be indoor sports spaces in girls’ schools.

## Conclusion and implication

Overall, the cultural and social conditions of the society have a great influence in determining the attitude, motivation, level of PL, and PA; therefore, since the findings of this research were based on Iran’s specific cultural and social environment, generalizations should be made with caution and the conditions mentioned should be taken into account. To determine more precisely the correlates in other cultural and social contexts, more studies are necessary for this area. In addition, the identification of PL correlates in girls and boys can contribute significantly to optimizing educational planning for students’ physical education courses. Ultimately, it is suggested that all individuals who are involved in developing the student PA should first know and understand the concept of PL. In fact, it is necessary for teachers, coaches, and other professionals who work with students to accept PL as a core goal. This is achieved by a deep understanding of the concept of PL in the context of social and ecological environments.

## Data Availability

Data would be available from the corresponding author on reasonable request.
